# Histone deacetylase signaling in cardioprotection

**DOI:** 10.1007/s00018-013-1516-9

**Published:** 2013-12-06

**Authors:** Lorenz H. Lehmann, Barbara C. Worst, David A. Stanmore, Johannes Backs

**Affiliations:** Research Unit Cardiac Epigenetics, Internal Medicine III, Heidelberg University and DZHK (German Center for Cardiovascular Research), partner site Heidelberg, Im Neuenheimer Feld 410, 69120 Heidelberg, Germany

**Keywords:** HDAC, Histone deacetylase, Heart failure, Cardiovascular therapeutics, Cardiovascular disease, HDAC inhibitors

## Abstract

Cardiovascular disease (CVD) represents a major challenge for health care systems, both in terms of the high mortality associated with it and the huge economic burden of its treatment. Although CVD represents a diverse range of disorders, they share common compensatory changes in the heart at the structural, cellular, and molecular level that, in the long term, can become maladaptive and lead to heart failure. Treatment of adverse cardiac remodeling is therefore an important step in preventing this fatal progression. Although previous efforts have been primarily focused on inhibition of deleterious signaling cascades, the stimulation of endogenous cardioprotective mechanisms offers a potent therapeutic tool. In this review, we discuss class I and class II histone deacetylases, a subset of chromatin-modifying enzymes known to have critical roles in the regulation of cardiac remodeling. In particular, we discuss their molecular modes of action and go on to consider how their inhibition or the stimulation of their intrinsic cardioprotective properties may provide a potential therapeutic route for the clinical treatment of CVD.

## Introduction

The modern health care systems in the Western world are currently faced with a dramatically increased prevalence of diseases related to the elderly, especially cardiovascular disease (CVD) [1]. In 2004, 13.7 % of the European population was aged 65 years or older. The mortality by CVD in Europe and North America is higher than 35 % [1, 2], making it the main cause of death in Europe and the USA [1, 3, 4]. Moreover, the economic burden of increasing CVD morbidity is enormous. In 2003, CVD cost the European Union €169 billion, the US €310 billion, and Canada €16 billion [4–6]. These calculations include direct costs, driven by patient-centered care, as well as indirect costs, driven by disability and increased mortality. A statement from the American Heart Association claims that more than 40 % of Americans are projected to have some form of CVD and direct medical costs are projected to triple, reaching over €870 billion in 2030 [7].

CVD represents a heterogeneous group of disorders and includes—as currently defined by the World Health Organization—arterial hypertension, coronary heart disease, cerebrovascular disease, peripheral arterial disease, heart failure, rheumatic heart disease, congenital heart disease, and cardiomyopathies [8]. The common serious consequence of CVD is a reduced systolic and/or diastolic cardiac function, which is driven by structural, cellular, and molecular changes. These changes initially counteract different cardiac stress situations but in the long run induce adverse cardiac remodeling, leading to cardiac dysfunction and ultimately to heart failure. Many preclinical therapeutic strategies are aimed at inhibiting these changes but until now none of these strategies have entered the clinical arena. Likewise, the development of such drugs seems to have become less attractive for pharmaceutical companies in recent years [9, 10]. Thus, it seems that novel therapeutic approaches are needed to prevent adverse cardiac remodeling. Adverse cardiac remodeling is driven not only by over-activation of detrimental signaling pathways but also by a loss of cardioprotective mechanisms. The stimulation of the latter would also be an attractive therapeutic strategy. However, much attention has been paid to the inhibition of adverse signaling cascades and not to stimulation of existing endogenous adaptive pathways.

In this review, we will highlight the role of certain cardioprotective mechanisms that are driven by a subset of chromatin-modifying enzymes, the so-called histone deacetylases (HDACs). In the past, a number of elegant mechanistic and mouse genetic studies have highlighted HDACs as key molecules for “adverse” signal transduction but recently we have begun to understand their role in molecular mechanisms that counteract adverse stress situations. The understanding of these “protective” mechanisms may help to develop novel therapeutic strategies.

## Histone deacetylases (HDACs)

Histone deacetylases were initially described as repressors of transcription [11]. They catalyze the removal of the acetyl groups from the amino side chains of lysine residues on histones, thereby restoring the positive charge, which results in stabilization of histone–histone and histone–DNA interactions. As a consequence, chromatin condensation occurs and access by transcription factors to their specific binding sites in the DNA is restricted. However, post-translational modification by acetylation and deacetylation does not occur solely to histones, but seems to be a fundamental mechanism by which other biological processes are regulated. In this regard, HDACs have also occasionally been referred to as ‘K’-DACs, pointing out a global role for lysine deacetylation not only in histones but also in non-histone proteins [12–14]. Accordingly, one should also take into account that HDACs/K-DACs may exert cardioprotective effects via indirect actions on transcription. For instance, an elegant study showed recently that HDAC4 can activate transcription by deacetylation of mitogen-activated kinase kinase kinase 2 (MEKK2) and consequent activation of the mitogen-activated kinase (MAP kinase) cascade [15]. The identification of additional non-histone target proteins will be a fascinating challenge for the future. However, relatively little work has been done in this arena and, therefore, these questions are not the focus of this review.

**Fig. 1:**
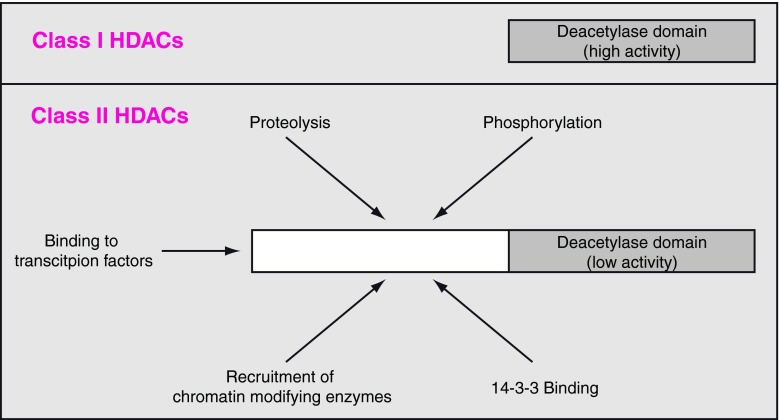
Basic structural difference of class I and class II HDACs. In contrast to class I HDACs, class II HDACs have a large N-terminal region. This allows an exposure to multiple cellular processes, such as modification by proteolysis, phosphorylation or recruitment of interacting proteins. Moreover class II HDACs function as scaffold proteins. In this context, they are able to bind to other proteins and chromatin modifiers (e.g., methyl transferases and class I HDACs). Moreover, via the N-terminus they are recruited to distinct transcription factors. The deacetylase activity is low compared to class I HDACs. *HDAC* histone deacetylase

Based on differences in their protein structure, the 18 mammalian HDACs are usually divided into four different classes. Whereas class I, II, and IV HDACs act in a Zn^2+^-dependent manner, class III HDACs act in a NAD-dependent manner. Class III HDACs are also called sirtuins and, as “K-DACs”, also regulate many non-histone proteins. This review focuses on the two classes of HDACs that have been most extensively studied with regard to their role in cardiac function: Class I (HDAC1, 2, 3 and 8) and class IIa (HDAC4, 5, 7 and 9). In contrast to class I HDACs, class IIa HDACs are exposed to multiple interactions and modifications, summarized in Fig. 1. We will also discuss the impact of available classical HDAC inhibitors (HDACi) as cardioprotective therapeutics and propose potential novel approaches to affecting HDAC function.

## Class I HDACs

Class I HDACs are ubiquitously expressed, localize preferentially to the nucleus, and possess high enzymatic activity toward histone substrates [16, 17]. They consist of HDAC1, 2, 3, and 8 and share significant homology to yeast retinoblastoma protein (Rpd3) [16, 18]. It was initially thought that these HDACs play a more general role in the regulation of gene transcription but mouse genetic studies conducted over the last 6 years have revealed distinct functions of class I HDACs with regard to cardiac function and pathology.

### HDAC1 and HDAC2

The first cardiac phenotype for mice lacking a class I HDAC was described by the Epstein lab [19]. HDAC2-deficient mice were created from a gene-trap embryonic stem cell line. These mice showed a partial lethality due to early myocardial defects. However, approximately 30 % of the mice survived and appeared to have a normal cardiac function in adulthood. When these HDAC2-deficient survivors were exposed to hypertrophic stimuli, cardiac hypertrophy and fibrosis were attenuated, indicating a detrimental role of HDAC2 upon pathophysiological conditions. Vice versa, cardiac-specific overexpression of HDAC2 resulted in cardiac hypertrophy, indicating that HDAC2 is not only required but also sufficient to drive maladaptive cardiac remodeling. Mechanistically, the authors could identify the inositol polyphosphate 5-phosphatase (Inpp5f) as a transcriptional target of HDAC2. Inpp5f seemed to inactivate rac protein kinase alpha (AKT), which in turn resulted in dephosphorylation and activation of the protein kinase glycogen synthase kinase 3β (GSK3β). GSK3β was confirmed as the critical downstream target because chemical inhibition of activated GSK3β allowed HDAC2-deficient adults to become sensitive to hypertrophic stimulation. Although the adaptive/maladaptive roles of GSK3β are not entirely understood and may depend on the type of cardiac damage, a large body of evidence suggests that GSK3β acts as a negative regulator of cardiac hypertrophy [20–23]. Thus, the authors suggested that inhibition of HDAC2 stimulates the anti-hypertrophic effects of GSK3β. This is of interest because it is more challenging to develop specific small compound activators of enzymes such as GSK3β than to develop specific inhibitors of the upstream HDACs. Conflicting results were reported by the Olson lab [24]. Montgomery and colleagues showed that mice in which HDAC2 had been globally deleted by homologous recombination, did not survive after birth and therefore could not be used to study its function for the adult heart under disease conditions. Instead, they generated conditional knockout mice, lacking HDAC2 only in cardiac myocytes. In contrast to Trivedi et al., these mice were not protected against cardiac hypertrophy induced by chronic β-adrenergic stimulation or pressure overload. Similarly, deletion of HDAC1 in cardiac myocytes failed to produce a protective effect against chronic β-adrenergic stimulation in mice, as did deletion of HDAC2 combined with a heterozygous deletion of HDAC1. Homozygous cardiac-specific deletion of HDAC1 and HDAC2 resulted in neonatal lethality, accompanied by cardiac arrhythmias and a phenotype resembling dilated cardiomyopathy. How might this apparent inconsistency be explained? Gene deletion by the gene-trap method, as used by Trivedi et al., often results only in a partial deletion of the gene, explaining why 30 % of the animals survived in this study [25]. Moreover, HDAC2 was deleted globally in the Trivedi study. Thus, it is possible that partial deletion of HDAC2 in non-cardiac myocytes such as cardiac fibroblasts might account for the protective effect. However, this interpretation is challenged by the observation that overexpression of HDAC2 in cardiac myocytes leads to the opposite phenotype. The recent finding that HDAC2 plays a major role in autophagy driven by α-adrenergic stimulation in cultured cardiac myocytes [26] provides another indication that HDAC2 may act as a driver of adverse cardiac remodeling. The true role of HDAC2 in the progression of CVD is therefore still unclear and future studies are warranted to answer this question.

### HDAC3

Whereas HDAC1 and 2 seem to play similar roles, the role of HDAC3 is different. Transgenic overexpression of HDAC3 in the heart leads to a hyperplasia phenotype [27]. Conditional knockout of HDAC3 in cardiomyocytes leads to cardiac hypertrophy and severe metabolic changes in the heart [28]. This was surprising because of the effects of other class I HDACs. HDAC3 is located in a complex called N-CoR (nuclear Co-Repressor) and SMRT (silencing mediator for retinoid and thyroid hormone receptors) that mediates transcriptional repression, which could explain in part the results of the cardiac-specific deletion [17]. By contrast, a later genetic deletion using a muscle creatine kinase (MCK)-dependent Cre lacked a cardiac phenotype under basal conditions but, when combined with a high-fat diet, led to a severe cardiac phenotype with lipid overload, heart failure, and highly increased mortality [29]. This was accompanied by downregulation of metabolic genes, although the specific mechanism by which HDAC3 deletion dysregulates metabolic and cardiac genes under these circumstances is not fully understood. Likewise, there is no evidence so far that a gain of HDAC3 function may exert beneficial effects on, for example, metabolic changes that are associated with heart failure. On the other hand, HDACi may have potentially harmful effects, as the inhibitors in use are mostly pan-specific and could therefore inhibit potentially beneficial effects of HDAC3.

### Open questions and conclusions

HDAC8-deficient mice show abnormalities in skull morphogenesis and die a few hours after birth [30]. A conditional cardiac deletion of HDAC8 and its consequence for the adult heart has not been investigated so far. The cardiac role of HDAC11 is presently unclear. Taken together, cardioprotective roles of class I HDACs have not been clearly demonstrated and harmful effects of their upregulation have been described. Thus, a gain of function approach with certain class I HDACs does not seem to be a good approach. The potential use of HDACi as cardioprotective approach is discussed in the next paragraph.

## HDAC inhibitors (HDACi)

**Fig. 2:**
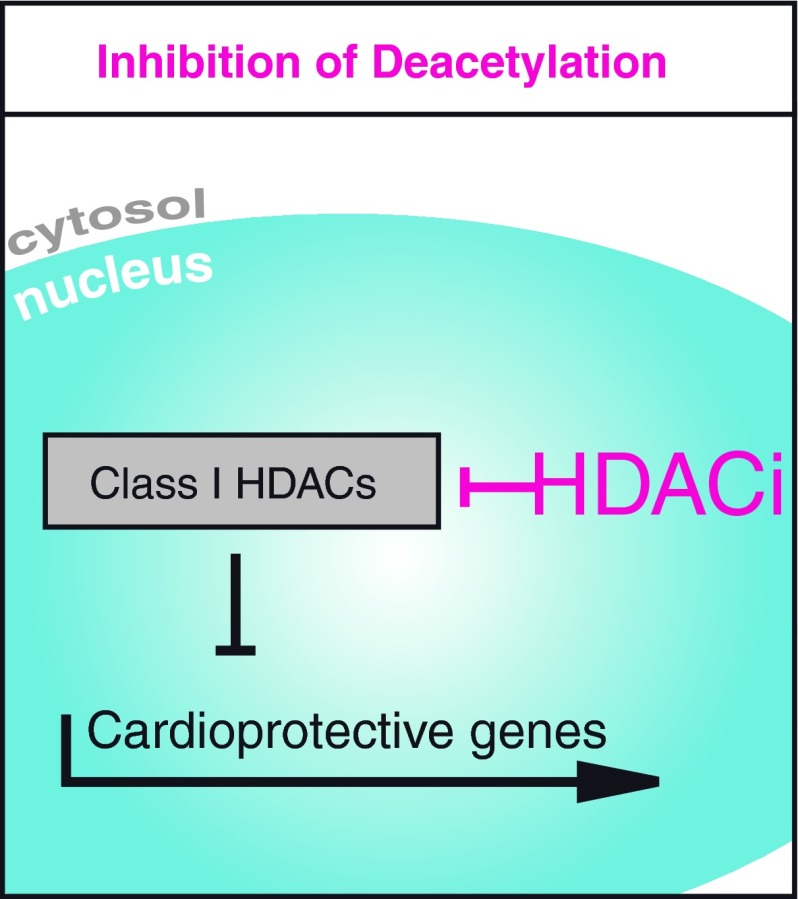
HDAC inhibitors (HDACi) target the deacetylation domain. HDACi are drugs with different specificities on HDACs. Because the deacetylase activity of class II HDACs is low, they primarily inhibit class I HDACs. Many of their functions are related to unspecific increase in acetylation activity within the nucleus and the cytoplasm. *HDAC* histone deacetylase, *HDACi* HDAC inhibitor

HDAC inhibitors consist of hydroxamic acids, benzamides, short-chain fatty acids and cyclic peptides, targeting the deacetylase domain of HDACs (Fig. 2). The specificity of inhibition depends on the assay and substrate used and is often debated. Because the class II HDACs have a relatively low intrinsic deacetylase activity [31, 32], it seems that the major targets of HDACi are class I HDACs. The hydroxamic acids SAHA and Trichostatin A (TSA) serve as pan-HDACi and are most commonly used for preclinical studies [31, 33–37]. Their effects are not solely observed on histone acetylation, but also on non-histone targets that are hyperacetylated after treatment [12, 13]. Furthermore, the short-chain fatty acid valproate is used in preclinical studies and has been in clinical use for the treatment of epilepsy since 1962 [34, 38]. HDACi were initially described as anti-proliferative drugs. Initial efforts to bring HDACi to the clinic originated from the cancer field and aimed to target highly proliferating cells to slow down tumor growth. SAHA (suberoylanilide hydroxamic acid, vorinostat), the first FDA approved drug to be specifically referred to as an ‘HDACi’ was provided for cutaneous T cell lymphoma (CTCL), a disease with high mortality and, thus far, unsatisfactory therapeutic options [39, 40]. There are currently more than 80 studies registered at clinicalTrials.gov that are recruiting patients treated with SAHA in different cancer diseases [41]. Romidepsine (FK-228) is only the second of the newer HDACi to be approved for use in humans, although there are currently more than ten additional compounds in advanced clinical testing [31]. HDACi exerts not only anti-proliferative but also anti-inflammatory effects [42, 43]. HDACi therapy has also been introduced in the treatment of HIV and was suggested as a strategy to target latently infected cells, which could serve as a potential approach for curative strategies [44].

### HDACi counteract cardiac hypertrophy

Here we will focus on HDACi as a potential therapeutic strategy for CVDs. This concept was introduced in 2003 when Antos and colleagues reported antihypertrophic effects of TSA in cultured neonatal rat cardiomyocytes [45]. This was an unexpected result at the time because data from the first HDAC knockout mice (see below) suggested the opposite. Nevertheless, these antihypertrophic effects were confirmed by others in vitro and in vivo [26, 34, 36, 45–47]. For example, in the same year, Kook et al. [36] found that cardiac hypertrophy of mice overexpressing the homeodomain-only protein (HOP) could be rescued by treatment with trichostatin A and valproic acid. Further in vivo evidence in mice indicated that HDACi treatment successfully blunted cardiac hypertrophy induced by isoproterenol (Iso), angiotensin (AngII) and pressure overload induced by arterial hypertension and transthoracic aortic banding [26, 34, 35, 48–50]. After myocardial infarction, HDACi led to an improved myocardial performance and reduced myocardial damage [33, 51–53]. In HOP transgenic mice, Kook et al. [36] provided evidence that the association of HDAC2 with the serum response factor (SRF) led to cardiac hypertrophy and fibrosis. Thus, HDAC2 was initially suggested as the critical target of HDACi with regard to cardioprotection, a hypothesis that was supported by the attenuated hypertrophic response in HDAC2-deficient mice as described above [19]. Initially, the cardioprotective effect was associated with increased expression of the phosphatase Inpp5f and its downstream activation of GSK3β. Later, other mechanisms by which HDACi might contribute to the antihypertrophic effects were also suggested. HDACi was shown to increase expression of the Krüppel-like-factor 4 (KLF4) [47, 54, 55] and KLF4-knockout animals developed exaggerated hypertrophy, whereas overexpression blunted cardiomyocyte hypertrophy in vitro.

### HDACi counteract cardiac fibrosis

In addition to antihypertrophic effects, HDACi were shown recently to have beneficial effects on fibrosis. Antifibrotic effects are present in in vivo models of hypertrophy. These effects are discussed in the context of reduced infarct size when animals are treated with HDACi [33, 35, 38, 51–53]. So far it is not entirely clear whether fibrosis is an active mechanism or just a replacement reaction, driven by a loss of cells resulting either from distinct non-necrotic cell death or from necrosis. The potential antifibrotic effects of HDACi in non-cardiac diseases are reviewed in detail elsewhere [56]. Briefly, antifibrotic effects are shown for renal fibrosis, diabetic nephropathy, idiopathic pulmonary fibrosis, cystic fibrosis and systemic sclerosis. Taken together, there is growing evidence that HDACi can directly target profibrotic pathways. Fibrosis is driven by fibroblasts, which, after stimulation by specific growth factors, differentiate to myofibroblasts and expresses alpha smooth muscle actin (αSMA). In human lung fibroblasts, the differentiation of fibroblasts to myofibroblasts was abolished by treatment with TSA [57]. In cardiac fibroblasts, TSA was able to inhibit collagen type I protein levels in a concentration-dependent manner [35]. Treatment with HDACi was also shown to have beneficial effects on other cellular processes that accompany pathological cardiac remodeling, namely the expression of tumor necrosis factor α, Interleukin-1β, nuclear factor-κB (NF-κB), reactive oxygen species (ROS) production and autophagy [26, 50]. HDACs are expressed not only in cardiomyocytes, but also in cardiac fibroblasts and endothelial cells [16, 58]. Therefore, it is not only a question of which HDAC, but also which cell type contributes to beneficial effects. However, well-defined genetic models have helped us to understand some basic mechanisms and they will also be of considerable use in the future.

### HDACi reduce ischemic injury

In the context of ischemia, TSA led to an increased resistance to ischemic injury, potentially via increased p38 activation [51]. Accordingly, p38 had previously been shown to translate the protective effects of late preconditioning in the heart [59]. In a follow-up paper, the group showed the involvement of the NF-κB pathway in this protective mechanism since NF-κB-knockout mice did not show a better outcome after TSA pretreatment. The induction of active caspase 3 was decreased and AKT phosphorylation was increased by TSA, accompanied by an increased angiogenic response within the same model [52]. Granger et al. [33] have shown the same effects in an I/R-model with prior intraperitoneal administration of TSA. By using RNAi to knockdown HDAC1–9 in neonatal rat cardiomyocytes (NRVMs), they show an induction of vascular endothelial growth factor (VEGF) that seems to be HDAC4-dependent. Furthermore VEGF was not induced in vivo after pretreatment with TSA. The authors conclude from these two findings, that HDAC4 may be the downstream target of HDACi pretreatment in I/R and that HDAC4 inhibits hypoxia inducible factor 1a (HIF1a) expression [33]. However, the in vivo role of this mechanism needs to be elucidated by further investigations. Taken together, HDACi treatment seems also to be beneficial in preclinical models for I/R and chronic ligation.

### HDACi and the heart in the clinical arena

Although the protective roles for HDACi in cardiac function, fibrosis and hypertrophy were extensively investigated in preclinical models, only one publication is currently known that addresses the question of whether HDACi have antihypertrophic effects in a clinical setting [60]. The authors did not find any effect of depsipeptide on cardiac mass in 12 months of treatment (echocardiography performed after 3, 6, and 12 months). The significance of the study is limited, since only ten individuals were included and echocardiography may not be sensitive enough to detect small changes in cardiac mass. Analysis of patients on HDACi therapy via cardiac MRI could be a promising approach to get reliable data on cardiac mass and function under HDACi therapy. Other clinical data from HDACi studies are focused on drug safety issues. Early on in the introduction of HDACi in the clinic for cancer treatment, there were reports of sudden cardiac death and cardiac arrhythmias [61]. ECG changes, such as T-wave flattening or inversion were observed in multiple trials [62]. In the drug safety study with romidepsin (LAQ824) on 32 patients, two patients showed QTc prolongation >500 ms accompanied by non-symptomatic ST-T-wave changes and one patient developed atrial fibrillation which led to a stop of therapy [63]. The patients received a relatively high dosage of HDACi (up to 100 mg/m^2^). Ejection fraction was assessed by multigated acquisition scan during the period of treatment for 3 weeks and showed no change in cardiac performance. Changes in cardiac troponin were not seen. Arrhythmias were only detected in patients with 72 and 100 mg/m^2^ [63]. LAQ824 was suggested to inhibit human Ether-à-go-go-Related Gene (hERG), a potassium ion channel that could explain the QTc prolongation after HDACi therapy. The proarrhythmic effect of HDACi therapy is questionable. Patients who participate in HDACi studies often have a high risk for arrhythmias driven by electrolyte disturbances. The study cohorts were often submitted to extensive and multiple therapies before HDACi, including stem cell transplantation and treatment with anthracyclines, which are known to cause cardiac damage. Furthermore, co-treatment with other drugs, such as antiemetics can cause arrhythmia [62]. Taken together, there is a need for substantial clinical data that belong to HDACi treatment and cardiovascular follow-up.

## Class II HDACs

Class II HDACs consists of HDAC4, 5, 6, 7, 9, and 10. Since there is a lack of substantial data for HDAC6 and HDAC10 for the cardiovascular field, we are focused in the following part on HDAC4, 5, 7, and 9. These HDACs are summarized as class IIa. In contrast to class I HDACs, class IIa HDACs consist of an N-terminal extension in addition to the deacetylase domain that is located at the C-terminus. The deacetylase activity of class IIa HDACs is much lower than the deacetylase activity of class I HDACs. A structural analysis of the deacetylase domain revealed a single amino acid exchange (tyrosine in class I HDACs versus a histidine in class IIa HDACs) to be responsible for this reduced enzymatic activity. A His-to-Tyr mutation (HDAC4 His-976-Tyr) led to a remarkable 1,000-fold increase in the histone deacetylase activity [32]. Moreover, knockout mice lacking only the deacetylase domain of HDAC4 but still expressing the N-terminal half (1–747) did not show the same developmental phenotype as mice lacking most of the N-terminal part [64, 65], indicating that the deacetylase domain of HDAC4 is dispensable for its biological function. Thus, the N-terminal half of class IIa HDACs is of great interest in regulating their function (Fig. 1). In this context, it should be pointed out that class II HDACs are able to crosstalk to class I HDACs and other potent chromatin-modifying genes, such as methyl transferases. Recently, it was shown that HDAC4 is able to silence the *nppb* promotor via such a crosstalk [66]. These changes in HDAC-dependent promoter methylation are relevant in patients with heart failure and suggest a novel diagnostic and perhaps therapeutic approach in the future.

### Class II HDACs in mouse models

Class IIa HDACs have been shown to regulate cardiac growth. Both HDAC5- and HDAC9-deficient mice showed no abnormal cardiac phenotype under unstressed conditions but developed excessive pathological cardiac growth in response to pressure overload evoked by transthoracic aortic banding [20, 67]. Conversely, overexpression of HDAC9 counteracted cardiomyocyte hypertrophy [20], but this has not yet been confirmed to occur in vivo. Mice lacking HDAC4 or HDAC7 are not viable and were therefore not investigated with regard to their function in the adult heart [65, 68]. HDAC4 null mice die soon after birth as a consequence of premature ossification and HDAC7 null mice die in utero due to reduced vascular integrity. Because of the potential cardioprotective functions of class IIa HDACs, many mechanistic studies were conducted over the last decade but there is an essential lack of in vivo data. It seems that a tight balance of class IIa HDACs seems to be essential for the cardioprotective function since overexpression of a signal-resistant HDAC5 mutant leads to early death due to severe heart failure [69].

### HDACs as repressors of transcription factors

Importantly, it has been shown that the N-terminal half of class IIa HDACs interacts and represses the activity of different transcription factors (TFs) including myocyte enhancer factor 2 (MEF2) [70–72], serum response factor (SRF) [73], nuclear factor of activated T-cells (NFAT) [74], calmodulin binding transcription activators (CAMTAs) [75] and GATA transcription factors [76]. These TFs are summarized in Table 1. Most of them are known to be important for myocyte differentiation during embryonic development [77–80] but have also been found to be upregulated in the adult heart during the development of heart failure (reviewed in [81–83]). The gene programs driven by these TFs can be regarded as an initial attempt by the heart to decrease wall stress and sustain appropriate circulation in response to cardiac stress but ultimately lead to adverse pathological remodeling [80, 84, 85]. With regards to the potential therapeutic use of class II HDACs or their manipulation for therapeutic approaches, it will be necessary in the future to identify the specific targets of distinct N-terminal regions. Our recently published data suggest that different N-terminal regions lead to differences in TF-binding and therefore to an inhibition of specific gene programs [86].

**Table 1:** Transcription factors that bind to class II HDACs

Class IIa HDACs shown to influence TF-activity	Interaction partner binding sites	Mouse model	Phenotype	Reference
TFs with increased activity in response to cardiac stress signaling—negatively regulated via class IIa HDACs				
MEF2				
HDAC4, HDAC5, HDAC7, MITR	HDAC4 163–180 HDAC5 175–192	MEF2A^-/-^	Not viable Lethal arrhythmias	[72, 77, 85, 146–148]
		MEF2C^-/-^	Not viable Impaired cardiac myogenesis	
		MEF2D^-/-^	Viable Resistant to remodeling after TAC, ISO	
		αMHC-MEF2A-Tg αMHC-MEF2C-Tg	Reduced cardiac function, enhanced by TAC But not by calcineurin overexpression	
NFAT				
HDAC4, HDAC5, HDAC7, HDAC9	Indirect via Mrj HDAC4 761–881	NFATc3^-/-^NFATc4^-/-^	Not viable Mitochondrial dysfunction Impaired cardiac development	[74, 80, 149, 150]
		NFATc3^-/-^	Viable Less hypertrophy after calcineurin, TAC, AngII	
		αMHC-NFAT3Δ317-Tg	Spontaneous hypertrophy, sudden death	
SRF				
HDAC4	HDAC4 201–289	SRF^-/-^	Not viable Defect in mesoderm formation	[73, 79, 84, 86, 151–154]
(HDAC5)	Indirect via myocardin?	βMHC-Cre:S^f^/S^f^	Not viable Impaired cardiac differentiation	
		αMHC-dmSRF	Early postnatal death Dilated cardiomyopathy	
		αMHC-MerCreMer:S^f^/S^f^	Die from heart failure	
		αMHC-SRF-Tg	Cardiomyopathy	
GATA				
HDAC4, HDAC5	Interact with and repress GATA1 in MEL cells	GATA4^-/-^	Not viable Severe cardiac defects	[76, 155–159]
		αMHC-Cre:GATA4^f^/GATA4^f^ βMHC-Cre:GATA4^f^/GATA4^f^ βMHC-Cre:GATA6^f^/GATA6^f^	Viable No hypertrophy, but apoptosis and Decompensation after TAC, AngII, PE	
		αMHC-GATA4-Tg	Cardiomyopathy	
CAMTA				
HDAC5, (HDAC4)	Link to Nkx2.5 HDAC5 153–360	CAMTA2^-/-^	Viable Less hypertrophic response to TAC, ISO, AngII	[75]
TFs with repressive activity on the fetal gene program—positively regulated via class IIa HDACs				
NRSF				
HDAC4, HDAC5	?	NRSF^-/-^	Not viable	[160–162]
		αMHC-dn-NRSF-TG	Dilated cardiomyopathy	
YY1				
HDAC5	?			[163]

Class II HDACs are repressors of different transcription factors. In case in vivo data were available that belong to cardiovascular phenotypes, we included the finding into the table

*HDAC* histone deacetylase, *TF* transcription factor, *MEF* myocyte enhancer factor, *NFAT* nuclear factor of activated T-cells, *SRF* serum response factor, *S* ^*f*^ SRF^f^, ^*f*^ floxed locus, *dmSRF* double mutant SRF (resulting in reduced binding to serum response elements), *GATA* *GATA* interacting TF, *CAMTA* calmodulin binding TF, *NRSF* neuron restrictive silencing factor, *dnNRSF* dominant negative mutant of NRSF, *YY1* yin-yang 1, *α/βMHC* α/β-myosin heavy chain, *ISO* isoproterenol, *TAC* transaortic constriction, *TG* transgene

### Signal-responsive nucleo-cytoplasmic shuttling of class II HDACs

Transcriptional repression by class II HDACs is strongly associated with their subcellular localization. HDAC4, HDAC5, HDAC7 and HDAC9 were found to have three conserved serine sites at the N-terminal domain (S246, 467 and 632 for HDAC4) [87]. Phosphorylation of at least one of them allows the association with the chaperone 14-3-3, exposing the C-terminal nuclear export signal [70] and inducing CRM-1-mediated (chromosome region maintenance 1) [88, 89] shuttling from the nucleus to the cytoplasm. Whereas exported HDACs lose their ability to repress MEF2 and other associated TFs, the triple mutant lacking the described phospho-sites is no longer signal-responsive and stays in the nucleus [87]. 14-3-3 binding is responsible for both nuclear export and inhibition of nuclear import [90]. Phosphorylation is a basic mechanism that leads to an export of class II HDACs to the cytosol but recently it has been shown that phosphorylation-independent mechanisms influence the cellular distribution of class IIa HDACs as well (see below). A simplified model of the regulation of class IIa HDACs in their role as transcriptional repressors would be as follows: Nuclear localization is associated with gene repression and export to the cytosol leads to gene activation. More provocatively, it might be said that export mechanisms promote heart failure whereas nuclear retention is cardioprotective. A variety of protein kinases have been reported to phosphorylate class IIa HDACs in order to induce translocation from the nucleus to the cytosol (reviewed in [83]), among them the Ca^2+^/calmodulin-dependent kinase II (CaMKII) and the protein kinase D (PKD). Both kinases are upregulated in the myocardium of mice as well as in humans during heart failure [91].

### Protein kinase D-dependent regulation of class II HDACs

PKD belongs to the CaMK superfamily with three different isoforms having more or less redundant effects on class IIa HDACs [92]. HDAC5 was identified early as a phosphorylation target of PKD [93], but HDAC4, 7 and 9 are also known to be targeted by PKD [94–96]. In vitro, PKD is activated after treatment with α-adrenergic receptor agonists and ET-1. Cardiac-specific deletion of PKD in turn leads to a protection from pro-hypertrophic stimuli (pressure overload via TAC, chronic β adrenergic stimulation with ISO, chronic AngII stimulation) [97]. The animals in this study showed lower heart weight/tibia length (HW/TL) ratios, less enhanced transcription of fetal genes and less fibrosis than WT mice. Cardiac-specific overexpression of PKD results in a dilated cardiomyopathy with elevated fetal genes that are normally controlled by MEF2, supporting the pathological role of PKD [98].

### CaMKII-dependent regulation of class II HDACs

In contrast to PKD, CaMKII selectively phosphorylates HDAC4 [99, 100], suggesting a central role for HDAC4 in the regulation of CaMKII-dependent gene expression. This effect can be attributed to a specific CaMKII docking site (HDAC4 585–608) that is missing in HDAC5, 7 and 9. Despite this, it should be noted that the formation of a complex between HDAC4 and HDAC5 allows CaMKII to phosphorylate HDAC5 and thereby facilitate its nuclear export [101]. Various hypertrophic agents such as adrenergic receptor (AR) agonists, AngII and endothelin (ET) enhance CaMKII activity by G-protein coupled receptor (GPCR) signaling and the increase of local intracellular Ca^2+^ levels [102, 103]. The consequences range from ion channel modification and alterations in Ca^2+^-handling to the stimulation of hypertrophic gene programs (reviewed in [103]). Transgenic mice with cardiac-specific overexpression of CaMKIIδ, the predominant isoform in the heart, show spontaneous hypertrophy, elevated MEF2 activity and the expression of embryonic genes, potentially explained by HDAC4 inactivation [104, 105]. Accordingly, global KO of CaMKIIδ is accompanied by normal development but a decreased hypertrophic response to pressure overload and lower levels of phosphorylated HDAC4 [106, 107]. The effect of CaMKIIδ deletion, however, is not as striking as expected, probably because of the overlapping functionality of CaMKIIγ, which can also be found in myocardial tissue. The creation of a CaMKIIγ/δ double-knockout could help to resolve this observation. However, taking into account that CaMKII specifically phosphorylates HDAC4, it could also an effect of partially redundant roles of class IIa HDACs within the heart.

### ROS-dependent regulation of class II HDACs

Abnormally high rates of ROS can be observed in cardiomyocytes in response to AngII or catecholamine signaling. ROS production contributes to cardiac hypertrophy (reviewed in [108, 109]). A lot of speculation has been made concerning possible downstream targets of ROS such as CaMKII, which has been observed to be activated via oxidation [110]. In an elegant study, Ago et al. [111] found a direct connection between redox state and HDAC4 localization. HDAC4 possesses two cysteines (C667,669), non-conserved among class IIa HDACs, that are oxidated upon phenylephrine (PE) treatment, a synthetic α-AR agonist. The oxidation leads to nucleocytoplasmatic shuttling of HDAC4 that can be prevented by either CRM-1 inhibition or treatment with the antioxidant thioredoxin 1 (Trx1). This phosphorylation-independent export of HDAC4 was shown to have the same pro-hypertrophic effects as the kinase-mediated ones [111, 112]. In accordance with ROS-dependent export of HDAC4, HDAC5 is also sensitive to ROS. Although PKD is recognized as the major modulator of HDAC5 activity, Haworth et al. [96] have shown that this pathway is less relevant in the presence of β-AR stimulation. They identified a new phosphorylation-independent, ROS-mediated mechanism to export HDAC5 after ISO treatment resulting in higher MEF2 activation levels. It would be worthwhile to also test the redox state of other class IIa HDACs, particularly HDAC4, in the scenario of enhanced β-AR signaling.

### PKA-dependent regulation of class II HDACs

**Fig. 3:**
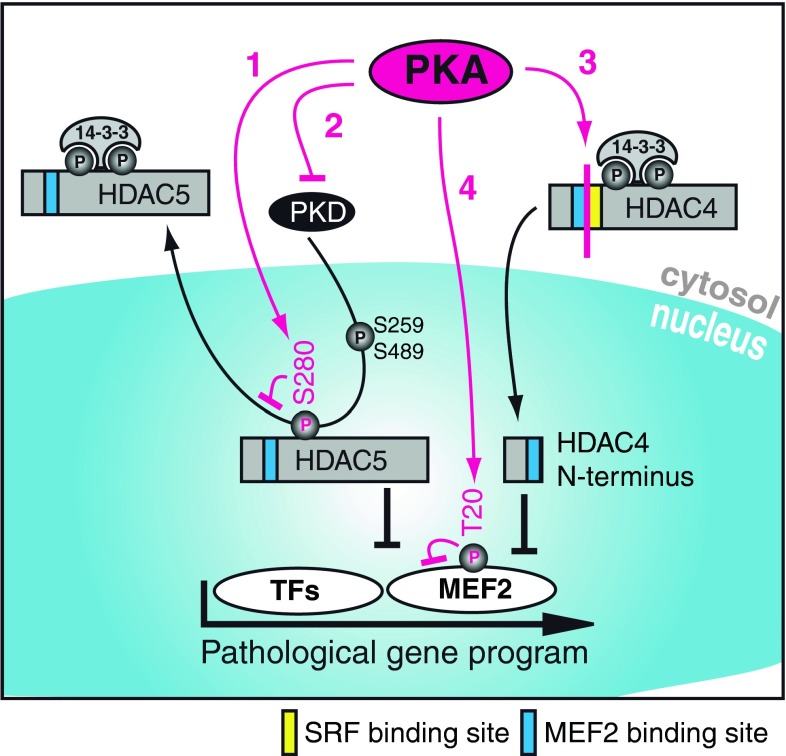
How protein kinase A (PKA) acts on class II HDACs. *1* PKA phosphorylates HDAC5 at S280 and inhibits 14-3-3 binding. *2* PKA inhibits protein kinase D (PKD) activation. *1+2* This results in nuclear accumulation of HDAC5 and consequent repression of transcriptional activity, thereby counteracting the phosphorylation of HDAC5 by PKD at S259 and S489, subsequent binding to the chaperon 14-3-3 and nuclear export (*black arrow* on HDAC5). *3* HDAC4 is cleaved by a PKA-dependent mechanism. The N-terminal cleavage product accumulates in the nucleus and inhibits myocyte enhancer factor 2 (MEF2). The cleavage takes place between binding sites for MEF2 and serum response factor (SRF), indicating that PKA leads to a shift in the affinity of HDAC4 towards MEF2 inhibition. *4* PKA is also able to directly inhibit MEF2 by phosphorylation at T20. All mechanisms lead to a reduced activation of hypertrophic transcription factors such as MEF2 and the related pathological gene program. *PKA* protein kinase A, *PKD* protein kinase D, *HDAC* histone deacetylase, *SRF* serum response factor

Protein kinase A (PKA) is a major downstream kinase of β-adrenergic receptors. Stimulation of this GPCR activates the adenylate cyclase (AC), which in turn leads to an increase in cyclic adenosine monophosphate (cAMP). cAMP is a second messenger with diverse cellular functions, including PKA activation. The duration of PKA activation is regulated by the phosphodiesterase (PDE)-dependent degradation of cAMP. The precise role of PKA in class IIa HDAC localization has started to be recognized very recently. Based on the existing data, it was difficult to judge whether PKA has beneficial or deleterious effects on the progression of heart failure. Recently, there has been growing evidence that PKA has a cardioprotective function, at least in the regulation of the induction of the fetal gene program. Moreover, recent data support the new idea that PKA activation plays an inferior role in heart failure [113]. In vitro, Haworth et al. showed that PKA activation via forskolin (a cAMP activator) or ISO treatment, or PDE inhibition is sufficient to prevent ET-1-induced PKD activation in adult rat ventricular myocytes (ARVM) [95, 114]. This mechanism is very likely able to reduce the PKD-dependent HDAC5 phosphorylation and thereby may inhibit derepression of MEF2. Under similar experimental conditions, enhanced PKA activity in response to cAMP, forskolin or ISO decreases the nuclear export of HDAC5 after treatment with ET-1 in NRVMs and ARVMs [115]. In contrast to other kinases, PKA-dependent phosphorylation of HDAC5 at serine 280 seems to result in decreased association with 14-3-3 and consequently decreased nuclear export. To summarize here, PKA works in two different ways on HDAC5: inhibition of PKD activity and phosphorylation at S280, both of which prevent derepression of MEF2 and other TFs. These data are still under discussion since the PKA-dependent phosphorylation of HDAC5 has not yet been reproduced in the work of other groups [96]. However, PKA itself was also demonstrated to directly phosphorylate MEF2D at serine 121 and 190 in skeletal myocytes in order to reduce transcription [116]. PKA signaling is not limited to HDAC5. Enhanced nuclear accumulation of HDAC4 can also be detected after PKA activation [116]. In accordance with these observations, our group has recently characterized a novel interaction between PKA and HDAC4. In response to stress signaling, an N-terminal cleavage product of HDAC4 (HDAC4-NT) is generated by a PKA-dependent proteolytic event in vitro and in vivo [86]. The cleavage site is located between amino acids 201 and 202, and is not present in other class IIa HDACs. This again highlights a central role for HDAC4 in β-adrenergic signal transduction. HDAC4-NT is sufficient to repress MEF2 to the same extent as HDAC4 full-length (HDAC4-FL), but has less impact on other TFs, especially on SRF, a critical TF for cell survival. By lacking the usual phosphosites, HDAC4-NT is no longer signal-responsive and stays in the nucleus. HDAC4-FL and another recently published caspase 3-induced HDAC4 cleavage product (HDAC4 2–289) seem to have pro-apoptotic effects when overexpressed in NRVM [117, 118]. Similar consequences were not detected for HDAC4-NT, possibly because of its reduced repression of SRF. This more or less specific repression of MEF2 by HDAC4-NT leads to an inhibition of the hypertrophic response to endothelin in vitro. We have highlighted the different PKA-dependent signaling pathways in Fig. 3. However, the question remains: how can these results be brought together with all of the other data denouncing PKA as a promoter of heart failure?

### Balance of kinases: “clock timer” for pathological remodeling

Based on the activation of β-AR, catecholamines, such as epinephrine and norepinephrine have positive effects on contractility and heart rate. This results in enhanced blood circulation to adequate levels during stress situations. It is therefore somewhat counterintuitive that, although initially contraindicated, β-AR antagonists or “β-blockers” are well-established drugs for the current “state of the art” therapy of patients with heart failure. This contradiction might be explained by the difference between chronic and acute adrenergic activation. Prolonged elevation of neurohormones during pathological stress situations turned out to have more negative than positive effects. Transgenic mice with cardiac-specific overexpression of β_1_ARs show increased contractility at an early age, but very soon their hearts become hypertrophic and insufficient [119, 120]. In the progression from a hypertrophic to a failing heart, the number of β-AR on the surface of myocytes decreases dramatically, which can be regarded as a self-protection mechanism [121, 122]. As mentioned above, the AC-cAMP-PKA pathway is also typically activated downstream of β-ARs. However, several studies have demonstrated that prolonged stimulation leads to a receptor desensitization with reduced PKA activity and enhanced CaMKII activation taking its place [113, 123, 124]. The analysis of the kinase activity in hearts from patients with idiopathic dilated cardiomyopathy (IDC) revealed an approximate three-fold increase in CaMKII but unchanged levels of PKA when compared to healthy patients [125]. Notably, cAMP levels are also decreased during heart failure [126]. This was recently supported by human data from patients with heart failure and hypertrophy, where CaMKII was activated in heart failure, but not PKA [113]. However, transgenic mice expressing the catalytic subunit of PKA at artificially high levels develop a severe dilated cardiomyopathy with hyperphosphorylation of PKA targets [127]. One of these targets is the ryanodine receptor (RyR), a Ca^2+^ channel in the membrane of the sarcoplasmic reticulum (SR) responsible for the Ca^2+^-triggered Ca^2+^ release during contraction. Phosphorylation in response to β-AR signaling enhances its Ca^2+^ sensitivity and therefore the contractility of the myocardium. However, prolonged stimulation is accompanied by hyperphosphorylation, which causes a Ca^2+^ leak, impaired excitation contraction (EC) coupling and thus reduced contractility as well as a disposition towards arrhythmias (reviewed in [128]). In fact, hyperphosphorylation of RyR at PKA sites is detectable in failing human hearts [129] and can be sufficiently inhibited by β-AR blockade [130, 131]. One possible explanation for this obviously increased PKA-mediated phosphorylation [although the catalytic PKA level is normal in the failing myocard (see above)], is a spatiotemporal difference in PKA activity at the cellular level. Special scaffolds called A-kinase anchoring proteins (AKAPs) form multiprotein complexes, which are comprised of PKA, other kinases, phosphatases, PDEs and further mediators to precisely couple receptor stimulation to intracellular processes [132, 133]. As a result of their activity, some pathways are up- and others downregulated, depending on the modalities of extra-cellular signals. It would be very interesting to investigate other PKA-mediated effects, especially the aforementioned repression of fetal gene expression under sustained β-AR stimulation with regard to the regulation via AKAPs.

In summary, under physiological conditions, β-AR stimulation is mainly coupled to the cAMP-PKA pathway, with the effect of enhanced contractility and the repression of fetal genes via HDAC4 cleavage and HDAC5 phosphorylation protecting the heart from pathological remodeling. Sustained elevation of catecholamines lead to a stimulation of the CaMKII pathway, leading to hypertrophy, remodeling and alterations in EC-coupling. In addition, a shift from “good” PKA effects to “bad” PKA effects may also contribute.

## Potential therapeutic strategies based on the regulation of class II HDACs

**Fig. 4:**
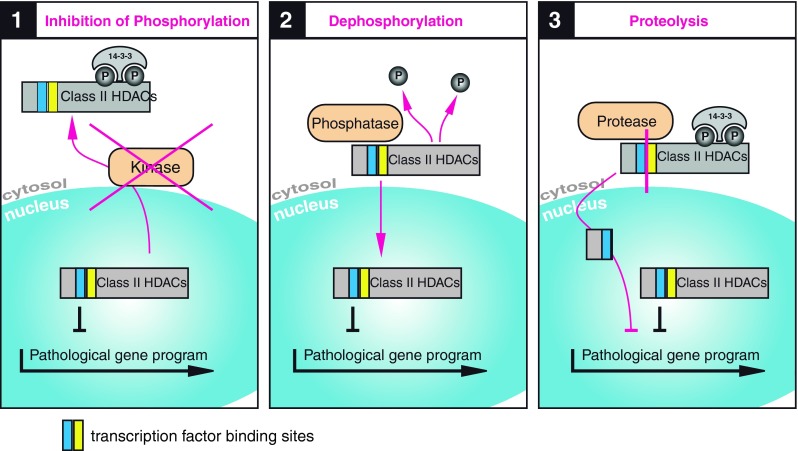
Therapeutic potential of class II HDACs: **1** Preventing nuclear export. Inhibition of phosphorylation could be achieved by small molecules that inhibit kinase activity or interrupt kinase-HDAC binding. Reduced phosphorylation would lead to reduced 14-3-3 binding with the consequence of nuclear accumulation of class II HDACs. This would result in inhibition of transcription, depending on the targeted kinase and targeted class II HDAC. **2** Enhancing nuclear import. Dephosphorylation of class II HDACs by activation of specific phosphatases leads to reduced 14-3-3 binding, to nuclear import and to nuclear accumulation of class II HDACs with the consequence of transcriptional repression. Phosphatases could be further investigated as potential drug target. **3** Induction of HDAC4 cleavage. Proteolysis of HDAC4, resulting in nuclear accumulation of the N-terminus leads to inhibition of MEF2. The advantage of this approach would be a more specific targeting of gene programs that are driven by MEF2, known to be active in pathological cardiac remodeling. So far, proteases in this pathway that could serve as potential drug targets are unknown. *HDAC* histone deacetylase

Which of the described pathways are crucial in the pathophysiology of human heart failure has to be further investigated. As there are still controversial data, it is hard to say what would be the perfect therapeutic approach. Simplified, one can say that class IIa HDACs act in a cardioprotective manner by preventing remodeling during normal stress situations with elevated catecholamines. Figure 4 suggests three possibilities: (1) prevention of nuclear export, (2) enhanced nuclear import or, according to the newly described PKA pathway, (C) induction of HDAC4 cleavage at Y201.

### Preventing nuclear export (Fig. 4-1)

Preventing the nuclear export of class IIa HDACs could, for example, be facilitated by inhibition of the CRM-1 export receptor, which was demonstrated to be anti-hypertrophic in cell culture. However, this approach seems inappropriate due to non-specific effects in vivo, as there are many other proteins shuttled by this receptor [134]. Kinase inhibition on the other hand is more specific. There are already data available about this mechanism. PKD inhibitor application in cell culture showed promising results [135]. However, the effects of orally administered inhibitors in animal models of HF were less impressive [136, 137]. Further drugs with different spectrum efficacies, e.g., those that also inhibit non-catalytic PKD effects, still need to be tested. Transgenic CaMKII inhibition was also shown to be cardioprotective during pathological β-AR stimulation and after MI [138, 139]. Application of the calmodulin antagonist W-7 reduces events of arrhythmias and improves left ventricular function in mice with atrioventricular block [140]. As CaMKII upregulation has multiple consequences, e.g., on both Ca^2+^ handling and transcriptional activation, it would be interesting to investigate the inhibition of the different pathways separately. One idea to selectively prevent the HDAC4 phosphorylation without influencing other CaMKII effects would be a small molecule that competes with HDAC4 for the binding at the CaMKII binding site. Over the past few years, there has been growing evidence for the pathophysiological role of ROS in the development of HF. Encouraging results have already been presented for the use of the antioxidant Trx1 in an ischemia–reperfusion mouse model, and there is growing evidence for the hypothesis that treatment with similar antioxidants may have beneficial effects on the progression of hypertrophy and remodeling (reviewed in [141, 142]).

### Enhancing nuclear import (Fig. 4-2)

Up to now, the export mechanisms of class IIa HDACs have been much better understood than the import mechanisms, so we can only speculate about therapeutic approaches. 14-3-3 is known not only to facilitate the export but also to prevent the import by binding to phosphorylated serine residues. 14-3-3 inhibitors, which are already being discussed as potential drugs for neurodegenerative diseases and cancer, may also have positive effects on heart diseases. Nevertheless, this would also be a fairly unspecific approach with unpredictable side effects that would require careful investigation. Upregulation or stimulation of HDAC phosphatases might be a promising approach, but they would first have to be identified and characterized. PP2A is one candidate, but the HDAC-specific regulatory subunits are not identified and other phosphatases may play additional roles [143, 144].

### Induction of HDAC4 cleavage (Fig. 4-3)

This pathway can be regarded as a physiological method of HDAC4 activation and gene repression in response to β-AR and PKA stress signaling. The short half-life time, the independence from kinase regulation and the absence of pro-apoptotic effects make us believe that this is perhaps a very promising pathway for therapeutic intervention. Several strategies are conceivable. PKA stimulation would probably be approached with high skepticism, due to the previously described adverse consequences of sustained PKA activation. From therapies with PDE-inhibitors we already know about the fatal side effects, such as arrhythmias and sudden death [145]. We therefore suggest pharmacological intervention further downstream in this pathway. Unfortunately, the serine protease cutting HDAC4 has not been identified yet, but it will certainly be an interesting drug target to investigate. Another idea is the overexpression of HDAC4-NT via gene therapy. This would be an elegant approach to selectively stimulate MEF2 repression without induction of apoptosis. Animal models will help to investigate whether the effects seen in cell culture are of clinical relevance. If so, HDAC4-NT overexpression in combination with, for example, β-AR blockade to improve EC coupling on the one hand and prevent remodeling on the other, would be interesting to test as a new strategy in HF therapy.
